# Thymoglobulin vs. ATG-Fresenius as Induction Therapy in Kidney Transplantation: A Bayesian Network Meta-Analysis of Randomized Controlled Trials

**DOI:** 10.3389/fimmu.2020.00457

**Published:** 2020-04-03

**Authors:** Turun Song, Saifu Yin, Xingxing Li, Yamei Jiang, Tao Lin

**Affiliations:** ^1^Department of Urology, Organ Transplantation Center, Institute of Urology, West China Hospital, Sichuan University, Chengdu, China; ^2^West China Medical School, Sichuan University, Chengdu, China

**Keywords:** thymoglobulin, ATG-Fresenius, kidney transplantation, induction therapy, network meta-analysis

## Abstract

**Background:** Thymoglobulin (THG) and antithymocyte globulin-Fresenius (ATG-F) have not been compared directly as induction therapies in kidney transplantation.

**Materials and Methods:** We performed a Bayesian network meta-analysis to compare THG with ATG-F by pooling direct and indirect evidence. Surface under the cumulative ranking curve (SUCRA) values were used to compare the superiority of one method over the other.

**Results:** A total of 27 randomized controlled trials (RCT) were eligible for the network meta-analysis. Efficacy endpoints, as well as safety indicators, were statistically comparable. For efficacy endpoints, THG seemed inferior to ATG-F in preventing delayed graft function [odds ratio (OR): 1.27; SUCRA: 78% vs. 58%], patient deaths (OR: 2.78; SUCRA: 83% vs. 34%), and graft loss (OR: 1.40; SUCRA: 83% vs. 59%), but superior to ATG-F in biopsy-proven acute rejection (BPAR; OR: 0.59; SUCRA: 78% vs. 39%) and steroid-resistant BPAR prevention (OR: 0.61; SUCRA: 76% vs. 49%) within the first year. For safety endpoints, THG was associated with higher risk of infection (OR: 1.49, SUCRA: 79% vs. 54%), cytomegalovirus infection (OR: 1.04; SUCRA: 40% vs. 37%), *de novo* diabetes (OR: 1.10; SUCRA: 90% vs. 30%), and malignancy (OR: 8.40; SUCRA: 89% vs. 6%) compared to ATG-F. A subgroup analysis of patients at high risk for immunologic complications revealed similar results, but THG performed better for graft loss (OR: 0.82; SUCRA: 68% vs. 54%).

**Conclusion:** ATG-F seemed to be more effective than THG in improving the short-term kidney transplantation outcomes. Prospective head-to-head comparison of THG and ATG-F with larger sample sizes and longer follow-up is still required.

## Introduction

Induction therapy with lymphocyte-depletion agents or lymphocyte-non-depletion agents that inhibit the T-cell response is widely used in kidney transplantation. Antithymocyte globulin (ATG), a T cell-depleting antibody, has been widely used for the prevention and treatment of acute rejection after kidney transplantation since the 1990s (1, 2). The two most commonly used preparations are rabbit ATG [thymoglobulin (THG); also known as ATG-Genzyme, Sanofi Genzyme, Cambridge, MA, USA] and ATG-Fresenius (ATG-F; also known as ATG-Grafalon, Fresenius Biotech GmbH, Munich, Germany). THG is derived from rabbits immunized with fresh human thymocytes, and ATG-F is produced from the Jurkat T lymphoblastic cell line (3, 4). Previous studies have shown that THG contains antibodies against a number of T-cell and B-cell antigens, including CD2, CD3, CD4, CD8, CD11, CD18, CD20, CD25, CD40, CD44, human leukocyte antigen DR (HLA-DR), and HLA class I (5), whereas the antibody profile of ATG-F is rarely against CD3, CD4, CD44, and HLA-DR but targeting CD28, CD29, CD45, CD49, CD98, and CD147 (6). Thus, THG and ATG-F may have different immunosuppressive activities.

Although THG and ATG-F have been prospectively compared with other induction therapies, including basiliximab, alemtuzumab, daclizumab, and muromonab-CD3 (OKT3), there is no head-to-head comparison between them. Previous retrospective analysis showed that THG induction is superior to ATG-F induction in preventing acute rejection and improving graft and patient survival (7, 8), and patients treated with ATG-F were less likely to have infections and malignancies (9, 10). Because of the retrospective nature of these studies, determining which ATG is better as an induction therapy for kidney transplantation is difficult.

Network meta-analysis, an extension of traditional pairwise meta-analyses, enables investigators to simultaneously pool data from the clinical comparisons of at least two treatments, and strengthens inferences about the relative efficacy of each treatment by including direct and indirect comparison information (11, 12). Hence, we used network meta-analysis to quantitatively synthesize the current evidence and compare the efficacy and safety of THG to ATG-F as an induction therapy in kidney transplantation.

## Materials and Methods

### Literature Search

All processes of the present study were conducted in accordance with the methods outlined in the Cochrane Handbook for Systematic Reviews of Interventions (13). A comprehensive literature search was first conducted by investigating PubMed, Embase, and the Cochrane Central Registry of Controlled Trials from their inception to January 22, 2019. We developed a specific search algorithm by combining MeSH terms and text words, including “thymoglobulin,” “antithymocyte globulin,” “ATG,” “ATG-Fresenius,” “ATG-F,” “kidney transplantation,” and “renal transplantation,” to identify all randomized controlled trials (RCTs). Accordingly, the researchers compared (1) the efficacy and safety of THG or ATG-F to other induction therapies and (2) the efficacy and safety of THG or ATG-F to no induction. There was no language or date restriction. We manually searched the reference lists of included studies for additional studies and the ClinicalTrials.gov registry for unpublished eligible trials.

### Inclusion Criteria and Study Selection

We included RCTs that met the following criteria: (1) patients receiving kidney transplants; (2) THG or ATG-F used as an induction therapy and compared with other induction options; (3) at least one of the following outcomes reported: delayed graft function (DGF), biopsy-proven acute rejection (BPAR), patient death, graft loss, renal function manifested in the creatinine serum level or estimated glomerular filtration rate, infection, cytomegalovirus (CMV) infection, *de novo* diabetes, and malignancy. Two reviewers (SFY and XXL) independently screened titles and abstracts after removing duplications and then obtained full texts for potential eligibility assessment. All discrepancies were resolved by consensus or with a third adjudicator (TRS).

### Data Extraction and Risk of Bias Assessment

The two reviewers used a predesigned form to extract information from eligible studies independently, including donor and recipient characteristics, induction therapy, and clinical outcomes of interest. The efficacy indicators were DGF, BPAR, patient death, and graft loss. The safety indicators were infection, CMV infection, *de novo* diabetes, and malignancy within the first year after kidney transplantation. The reviewers independently assessed the included studies for bias using the Cochrane Collaboration risk-of-bias tool (14), and any disagreement was resolved by consensus or with the third adjudicator.

### Statistical Analysis

We used a traditional pairwise meta-analysis to compare THG or ATG-F with other induction therapies. We used Review Manager version 5.3 (Nordic Cochrane Centre, Cochrane Collaboration, Copenhagen, Denmark) to perform this. Then, we conducted a Bayesian network meta-analysis to compare all outcomes between THG and ATG-F using the GeMTC Package in R version 3.5 (R Foundation for Statistical Computing, Vienna, Austria).

First, we examined evidence patterns for each outcome in the network plots to determine whether we could form closed loops, which is the nature of network meta-analysis. In these network plots, node and connection size correspond to the number of patients and studies, respectively.

Then, we conducted pairwise meta-analyses using random-effect models. Odds ratios (ORs) and 95% confidence intervals (CIs) were calculated. Heterogeneity was checked with the Q-statistic and the *I*^2^ test. Levels of *P* < 0.05 or *I*^2^ > 50% indicated the existence of heterogeneity.

In addition, we performed Bayesian network meta-analyses. The consistency and inconsistency estimates of THG vs. ATG-F are reported as ORs with 95% CIs. The relative ranking probabilities of all outcomes were also generated to calculate the values of the surface under the cumulative ranking curve (SUCRA) and explore the probability that one method would have superior specific endpoints. Higher SUCRA value indicates that a given treatment is more likely to be in the top rank or highly effective, and a value of “0” means that the treatment must be the worst. The corresponding degree of direct and indirect results was assessed by the node-splitting method (14).

We also performed a series of subgroup analyses to test the robustness of our results. First, to assess reliability, we compared the overall estimates with single estimates using different induction therapies as intermediaries. Second, we conducted a subgroup analysis of studies involving patients of high or low immunological risk to better reflect real-world scenarios. High immunological risk patients refers to those who have higher risk of developing AR and graft loss after transplantation, including patients with PRA > 20%, or re-transplant, or those who are Africa-American (15, 16). Third, we conducted the subgroup analysis by excluding the sensitive studies displayed in the funnel plots. Network funnel plots were generated by Stata 14 (StataCorp LLC, College Station, TX, USA) to assess the publication bias.

## Results

### Study Selection

**Figure 1:**
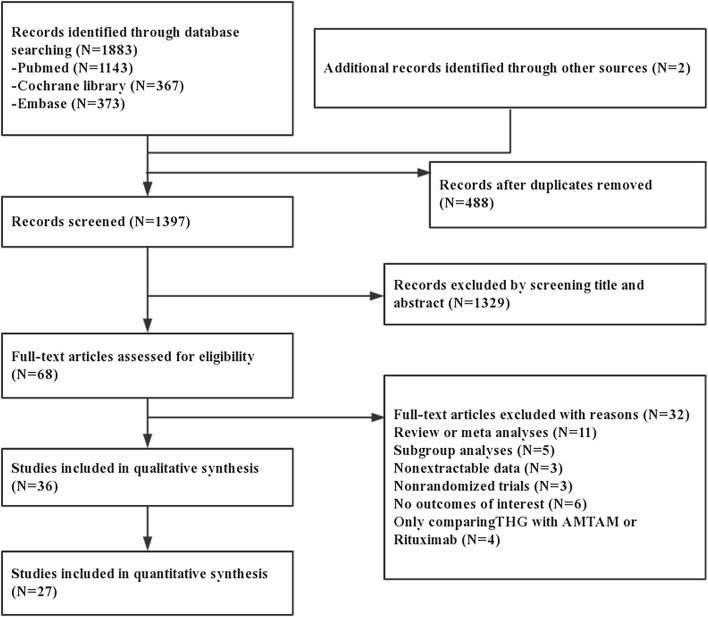
Systematic search and screening process of trials. ATGAM, equine-derived ATG.

The details of our literature search are shown in Figure 1. Of the 1,885 potentially relevant articles, 488 were excluded after duplications screening, and 1,329 were removed after the titles and abstracts were screened. Of the remaining 68 full-text articles, 32 were removed after full-text screening; thus, 36 studies were included in the qualitative analysis, and 27 trials were used in the quantitative analysis (15–41).

### Study Characteristics

The key characteristics of patients from 27 trials are summarized in Table 1 and Table S1. These studies were published from 1994 to 2016, and the sample size varied from 21 to 381. In 10 trials, the investigators compared ATG-F with other induction therapies (basiliximab in two studies, no induction in five, alemtuzumab in one, daclizumab in one, and muromonab-CD3 in one). In 16 trials, the investigators compared THG with other induction therapies (basiliximab in six, no induction in two, alemtuzumab in five, daclizumab in two, and muromonab-CD3 in one). In one trial, the investigators compared THG and ATG-F. The follow-up time varied from 12 to 60 months. Nine studies included patients at high risk for immunologic complications. In 14 of the 15 studies of THG, THG was administered in divided doses; in 11 of the 12 studies of ATG-F, ATG-F was administered in a single dose.

**Table 1:** Key characteristics of 27 included studies in the network meta-analysis.

**References**	**Country**	**Follow-up period**	**Arm**	**Size**	**Donor information**			**Recipient information**							
					**Mean age (years)**	**Sex (Male) (%)**	**Resource (deceased) (%)**	**Mean age (years)**	**Sex (Male) (%)**	**Race (White) (%)**	**Time on dialysis (month)**	**Mean HLA**	**Mean cold ischemic time (hour)**	**Immunological risk status**	**Dose information**
Thomusch et al. (15)	Germany	12 months	Basiliximab	189	55.0	/	89.0%	54.0	65.0%	98.0%	/	/	11.7	Low	20 mg on day 0 and day 4
			Thymoglobulin	192	53.1	/	88.0%	53.6	65.0%	98.0%	/	/	12.2		1.5 mg/kg for 4 days
Burkhalter et al. (16)	Switzerland	24 months	ATG-F	18	55.0	50.0%	78.0%	55.0	61.0%	/	/	3.8	7.3	High	day 0: 9 mg/kg; days 1–4: 3 mg/kg/day
			ATG	17	57.0	65.0%	71.0%	52.0	59.0%	/	/	2.9	7.5		day 0: 1.5 mg/kg; days 1–3: 1.5 mg/kg
Tedesco et al. (17)	Brazil	12 months	Thymoglobulin	85	39.9	61.0%	78.8%	43.7	63.0%	48.0%	37.1	2.6	21.4	Low/moderate	day 0: 3 mg/kg
			Basiliximab	102	42.6	57.0%	64.7%	45.1	67.0%	50.0%	42.2	2.7	20.6		20 mg on day 0 and day 4
Pilch et al. (18)	American	12 months	Basiliximab	98	/	/	81.0%	49.0	63.0%	49.0%	/	/	/	Low/high	days 0 and 4: 20 mg
			Thymoglobulin	102	/	/	81.0%	52.0	58.0%	50.0%	/	/	/		1.5 mg/kg for days 0, 1, 2, 3, 4
van den Hoogen et al. (19)	Netherlands	3 months	ATG-F	28	/	/	100.0%	54.0	64.0%	/	/	/	16.4	/	A single, high dose (9 mg/kg)
			No induction	24	/	/	100.0%	56.0	71.0%	/	/	/	16.6		/
Lu et al. (20)	China	338 days	Alemtuzumab	11	/	/	/	38.9	45.5%	/	/	/	/	High	A single dose (15 mg)
			ATG-F	11	/	/	/	40.8	36.4%	/	/	/	/		A single, high dose (9 mg/kg)
Hanaway et al. (21)	American	36 months	Alemtuzumab	70	33.7	59.0%	60.0%	44.7	53.0%	26.0%	/	/	12.0	High	A single dose (30 mg)
			Thymoglobulin	69	36.9	49.0%	62.0%	48.5	57.0%	29.0%	/	/	13.1		Days 0, 1, 2, and 3 or 4: 1.5 mg/kg
Ciancio et al. (22)	American	36 months	Thymoglobulin	13	39.2	/	/	44.5	76.9%	53.8%	/	2.9	/	/	1 mg/kg/day for 7 days
			Alemtuzumab	13	42.2	/	/	40.0	69.2%	38.5%	/	2.9	/		0.3 mg/kg/day for 5 days
Noel et al. (23)	France	12 months	Thymoglobulin	113	44.3	67.3%	/	45.4	46.0%	/	/	/	24.0	High	1.25 mg/kg/day for 7 days
			Daclizumab	114	44.6	57.0%	/	46.9	51.8%	/	/	/	22.7		1 mg/kg on days 0, 14, 28, 42, and 56
Farney et al. (24)	American	24 months	Alemtuzumab	113	40.0	/	65.0%	51.0	59.0%	65.0%	/	3.4	/	/	A single dose (30 mg)
			Thymoglobulin	109	43.0	/	72.0%	49.0	57.0%	63.0%	/	3.4	/		1.5 mg/kg for days 0, 2, 4…
Sheashaa et al. (25)	Egypt	60 months	ATG-F	40	/	/	/	30.3	82.5%	%	21.6	/	/	/	A single high-dose (9 mg/ kg)
			No induction	40	/	/	/	31.7	82.5%		21.6	/	/		/
Samsel et al. (26)	Poland	60 months	ATG-F	40	40.4	60.0%	100.0%	43.0	57.5%	/	34.0	3.3	30.4	/	A single, high dose (9 mg/kg)
			No induction	39	37.3	83.3%	100.0%	40.0	64.1%	/	38.0	3.1	30.4		/
Kim et al. (27)	Switzerland	24 months	ATG-F	11	37.0	55.0%	100.0%	52.0	18.0%	/	/	3.5	18.3	High	A single high-dose (9 mg/ kg)
			Daclizumab	11	46.0	73.0%	90.0%	51.0	36.0%	/	/	2.9	14.3		1 mg/kg on days 0, 14, 28, 42, and 56
Cantarovich et al. (28)	France	240 months	No induction	63	/	/	100.0%	40.0	74.6%	/	/	2.4	38.0	/	/
			Thymoglobulin	60	/	/	100.0%	36.0	68.3%	/	/	2.6	39.0		/
Abou-Ayache et al. (29)	France	12 months	Daclizumab	54	40.0	/	100.0%	44.0	70.0%	96.0%	28.0	2.0	18.0	/	2 mg/kg on day 0; 1 mg/kg on day 14
			Thymoglobulin	55	42.0	/	100.0%	45.0	69.0%	96.0%	23.0	1.6	20.0		1–1.5 mg/kg
Thomas et al. (30)	American	377 days	Alemtuzumab	11	/	/	/	43.5	54.5%	45.1%	/	/	/	High	A single dose (30 mg)
			Thymoglobulin	10	/	/	/	47.1	20%	10.0%	/	/	/		1.5 mg/kg on days 0, 1, 2, 3, 4
Kyllönen et al. (31)	Finland	60 months	ATG-F	53	40.6	/	/	47.8	26.4%	/	16.7	/	21.8	High	A single, high dose (9 mg/kg)
			Basiliximab	58	42.0	/	/	45.5	46.6%	/	7.9	/	22.7		20 mg on day 0 and day 4
			No induction	44	40.0	/	/	47.5	34.1%	/	9.0	/	22.1		/
Hernandez et al. (32 )	Spain	24 months	Thymoglobulin	80	45.0	/	52.5%	47.0	73.8%	/	20.0	3.4	20.3	Low	1–1.5 mg/kg/day for 7 days
			Basiliximab	80	42.0	/	62.5%	48.0	62.5%	/	24.0	3.7	21.0		20 mg on days 0 and 4
Brennan et al. (33)	/	12 months	Thymoglobulin	141	46.8	53.9%	/	51.3	56.0%	60.3%	/	/	25.4	High	1.5 mg/kg for days 0, 1, 2, 3, 4
			Basiliximab	137	46.9	62.8%	/	49.7	59.9%	65.0%	/	/	27.1		20 mg on days 0 and 4
Ciancio et al. (34)	Amertican	12 months	Thymoglobulin	30	33.4	/	100.0%	49.3	63.3%	50.0%	/	2.0	33.0	/	1 mg/kg for a 7-day course
			Alemtuzumab	30	35.9	/	100.0%	50.2	63.3%	33.3%	/	1.7	32.2		0.3 mg/kg on day 0 and day 4
Mourad et al. (35)	France	12 months	Basiliximab	52	42.4	/	96.2%	45.3	57.7%	/	/	/	/	Low	20 mg on days 0 and 4
			Thymoglobulin	53	43.4	/	98.1%	45.4	60.4%	/	/	/	/		1 mg/kg on day 0 and day 1
Tullius et al. (36)	Germany	12 months	ATG-F	62	42.0	/	100.0%	48.0	56.5%	/	79.0	/	14.0	/	A single high-dose (9 mg/ kg)
			Basiliximab	62	41.0	/	100.0%	48.0	53.2%	/	83.0	/	14.0		20 mg on days 0 and 4
Lebranchu et al. (37)	France	12 months	Basiliximab	50	41.1	/	100.0%	44.1	72.0%	92.0%	27.0	3.5	19.3	/	20 mg on days 0 and 4
			Thymoglobulin	50	41.5	/	100.0%	45.8	64.0%	94.0%	19.2	3.5	20.4		1 mg/kg on day 0 and day 1
Yussim and Shapira (38)	Israel	24 months	ATG-F	19	/	/	/	/	/	/	/	/	/	/	A single high-dose (9 mg/ kg)
			No induction	19	/	/	/	/	/	/	/	/	/		/
Thibaudin et al. (39)	France	25 months	No induction	42	35.0	/	/	46.0	29.0%	/	/	2.4	29.0	High	/
			Thymoglobulin	47	39.0	/	/	47.0	40.0%	/		2.5	/		1.25 mg/kg/day for 7 days
Bock et al. (40)	Switzerland	12 months	ATG-F	53	/	/	86.8%	46.0	66.0%	/	/	2.2	15.4	/	4 mg/kg for 7 days or 14 days
			OKT3	51	/	/	86.3%	49.0	58.8%	/	/	2.3	15.9		5 mg for 7 days or 14 days
Cole et al. (41)	Canada	12 months	Thymoglobulin	83	/	/	100.0%	48.4	66.0%	/	22.0	1.3	32.6	/	0.15 ml/kg/day for 10–14 days
			OKT3	83	/	/	100.0%	47.3	60.0%	/	24.0	1.4	32.9		Unknown doses for 10–14 days

*ATG, anti-thymoglobulin; ATG-F, anti-thymoglobulin Fresenius; DGF, delayed graft function; CMV, cytomegalovirus; OR, odds ratio; 95%CI, 95% confidence intervals; NS, not significant; NA, not available; BPAR, biopsy-proved acute rejection*.

### Study Quality

The quality assessment of included trials is presented in Table S2. Of the 27 RCTs, 23 (85%) exhibited low risk of bias in a random sequence generation: 16 (59%) in allocation concealment, 11 (41%) in “blinding” participants and personnel, 3 (11%) in “blinding” the outcome assessment, 21 (78%) in incomplete outcome data, and 23 (85%) in selective reporting. Overall, 19 RCTs (70%) were free of bias in all the domains just mentioned.

### Network Plot

In most studies, the investigators reported first-year outcomes. The network evidence patterns of all endpoints of interest are displayed in Figure S1. For the efficacy indicators, the network of the DGF included seven arms, 18 studies, and 2,057 patients; the network of BPAR included nine arms, 22 studies, and 2,744 patients; the network of steroid-resistant BPAR included two arms, five studies, and 976 patients; the network of patient death included seven arms, 17 studies, and 2,416 patients; and the network of graft loss included nine arms, 22 studies, and 2,540 patients. For the safety indicators, the network of infection included seven arms, 14 studies, and 1,960 patients; the network of CMV infection included three arms, 10 studies, and 1,681 patients; the network of *de novo* diabetes included two arms, four studies, and 776 patients; and the network of malignancy included two arms, five studies, and 976 patients. Of these nine network plots, six had a closed loop, and the other three (those for steroid-resistant BPAR, *de novo* diabetes, and malignancy) had an open loop.

### Results of the Pairwise Meta-Analysis

The results of pairwise comparisons are displayed in Table 2. Compared to no induction, THG was associated with reduced risk of DGF (OR: 0.56; 95% CI: 0.32 to 1.00), BPAR (OR: 0.34; 95% CI: 0.15 to 0.82), and graft loss (OR: 0.32; 95% CI: 0.14 to 0.74). In addition, THG contributed to reducing the incidence of BPAR in comparison with basiliximab (OR: 0.70; 95% CI: 0.50 to 0.97) and muromonab-CD3 (OR: 0.35; 95% CI: 0.19 to 0.67). ATG-F reduced the incidence of DGF in comparison with basiliximab (OR: 0.19; 95% CI: 0.05 to 0.70) but increased the risk of BPAR in comparison with muromonab-CD3 (OR: 2.42; 95% CI: 1.05 to 5.55).

**Table 2:** Results of pair wise meta-analyses.

**Outcomes**	**Pair-wise**	**Study number (effective/available)**	**OR (95%CI)**	***I*^**2**^**	***P***
DGF	Thymoglobulin (vs. basiliximab)	5/5	0.93 (0.71–1.24)	0.0%	NS
	ATG-Fresenius (vs. basiliximab)	1/1	0.19 (0.05–0.70)	NA	NA
	Thymoglobulin (vs. no induction)	2/2	0.56 (0.32–1.00)	0.0%	NS
	ATG-Fresenius (vs. no induction)	3/3	0.77 (0.20–2.93)	74.6%	<0.05
	Thymoglobulin (vs. daclizumab)	3/4	0.75 (0.43–1.32)	22.5%	NS
	ATG-Fresenius (vs. daclizumab)	1/1	6.00 (0.53–67.65)	NA	NA
	Thymoglobulin (vs. ATG-Fresenius)	1/1	1.92 (0.43–8.33)	NA	NA
BPAR	Thymoglobulin (vs. basiliximab)	7/7	0.70 (0.50–0.97)	0.0%	NS
	ATG-Fresenius (vs. basiliximab)	2/2	1.09 (0.58–2.04)	0.0%	NS
	Thymoglobulin (vs. no induction)	1/1	0.34 (0.15–0.82)	NA	NA
	ATG-Fresenius (vs. no induction)	3/3	0.63 (0.11–3.64)	NA	NA
	Thymoglobulin (vs. alemtuzumab)	4/5	1.60 (0.95–2.68)	0.0%	NS
	ATG-Fresenius (vs. alemtuzumab)	1/1	0.90 (0.10–7.78)	NA	NA
	Thymoglobulin (vs. OKT3)	1/1	0.35 (0.19–0.67)	NA	NA
	ATG-Fresenius (vs. OKT3)	1/1	2.42 (1.05–5.55)	NA	NA
	Thymoglobulin (vs. ATG-Fresenius)	1/1	1.72 (0.25–12.50)	NA	NA
Steroid-resistant BPAR	Thymoglobulin (vs. basiliximab)	3/3	0.53 (0.23–1.21)	0.0%	NS
	ATG-Fresenius (vs. basiliximab)	2/2	0.81 (0.21–3.12)	0.0%	NS
Patient death	Thymoglobulin (vs. basiliximab)	6/7	0.84 (0.44–1.61)	0.0%	NS
	ATG-Fresenius (vs. basiliximab)	0/2	NA	NA	NA
	Thymoglobulin (vs. alemtuzumab)	2/4	1.45 (0.45–4.76)	0.0%	NS
	ATG-Fresenius (vs. alemtuzumab)	1/1	2.00 (0.16–25.00)	NA	NA
	Thymoglobulin (vs. OKT3)	1/1	1.14 (0.42–3.13)	NA	NA
	ATG-Fresenius (vs. OKT3)	1/1	0.46 (0.08–2.63)	NA	NA
	Thymoglobulin (vs. ATG-Fresenius)	1/1	1.72 (0.22–12.5)	NA	NA
Graft survival	Thymoglobulin (vs. basiliximab)	6/6	0.91 (0.58–1.42)	0.0%	NS
	ATG-Fresenius (vs. basiliximab)	2/2	0.37 (0.09–1.44)	0.0%	NS
	Thymoglobulin (vs. no induction)	2/2	0.32 (0.14–0.74)	0.0%	NA
	ATG-Fresenius (vs. no induction)	4/4	0.72 (0.23–2.26)	0.0%	NS
	Thymoglobulin (vs. alemtuzumab)	3/3	1.03 (0.40–2.67)	0.0%	NS
	ATG-Fresenius (vs. alemtuzumab)	1/1	2.00 (0.16–25.75)	NA	NA
	Thymoglobulin (vs. OKT3)	1/1	1.16 (0.55–2.47)	NA	NA
	ATG-Fresenius (vs. OKT3)	1/1	0.38 (0.12–1.18)	NA	NA
	Thymoglobulin (vs. ATG-Fresenius)	0/1	NA	NA	NA
Infection	Thymoglobulin (vs. basiliximab)	6/6	1.04 (0.54–2.02)	82.0%	<0.05
	ATG-Fresenius (vs. basiliximab)	2/2	0.58 (0.31–1.10)	0.0%	NS
	Thymoglobulin (vs. no induction)	2/2	0.85 (0.25–2.94)	12.5%	NS
	ATG-Fresenius (vs. no induction)	1/1	0.58 (0.10–3.54)	NA	NA
	Thymoglobulin (vs. OKT3)	1/1	0.66 (0.11–4.05)	NA	NA
	ATG-Fresenius (vs. OKT3)	1/1	0.31 (0.03–3.06)	NA	NA
	Thymoglobulin (vs. ATG-Fresenius)	1/1	0.83 (0.20–3.49)	NA	NA
CMV infection	Thymoglobulin (vs. basiliximab)	7/7	1.25 (0.64–2.38)	78.5%	<0.05
	ATG-Fresenius (vs. basiliximab)	2/2	1.75 (0.55–5.56)	37.9%	NS
	Thymoglobulin (vs. ATG-Fresenius)	1/1	2.44 (0.39–16.67)	NA	NA
*De novo* diabetes	Thymoglobulin (vs. basiliximab)	2/2	0.98 (0.62–1.56)	0.0%	NS
	ATG-Fresenius (vs. basiliximab)	2/2	0.38 (0.10–1.47)	0.0%	NS
Malignancies	Thymoglobulin (vs. basiliximab)	4/4	1.79 (0.86–3.70)	0.0%	NS
	ATG-Fresenius (vs. basiliximab)	1/1	0.35 (0.03–3.45)	NA	NA

*ATG, anti-thymoglobulin; ATG-F, anti-thymoglobulin Fresenius; DGF, delayed graft function; CMV, cytomegalovirus; OR, odds ratio; 95% CI, 95% confidence intervals; NS, not significant; NA, not available; BPAR, biopsy-proved acute rejection*.

### Results of the Network Meta-Analysis

**Figure 2:**
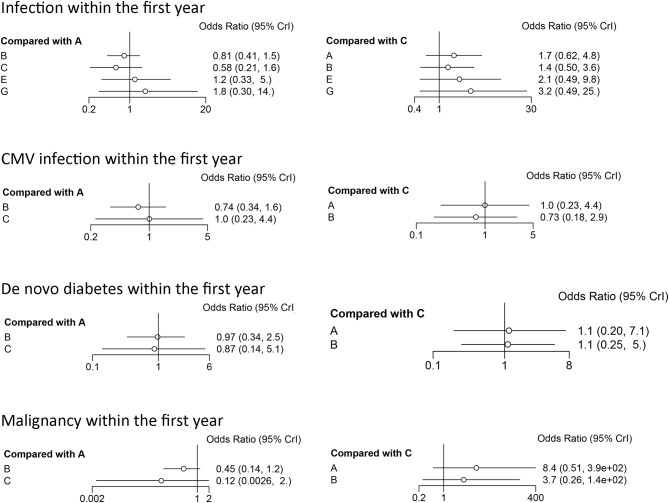
Forest plots of efficacy indicators. DGF, delayed graft function; BPAR, biopsy-proved acute rejection; A, thymoglobulin; B, basiliximab; C, ATG-Fresenius; D, no induction therapy; E, alemtuzumab; F, daclizumab; G, OKT3.

**Figure 3:**
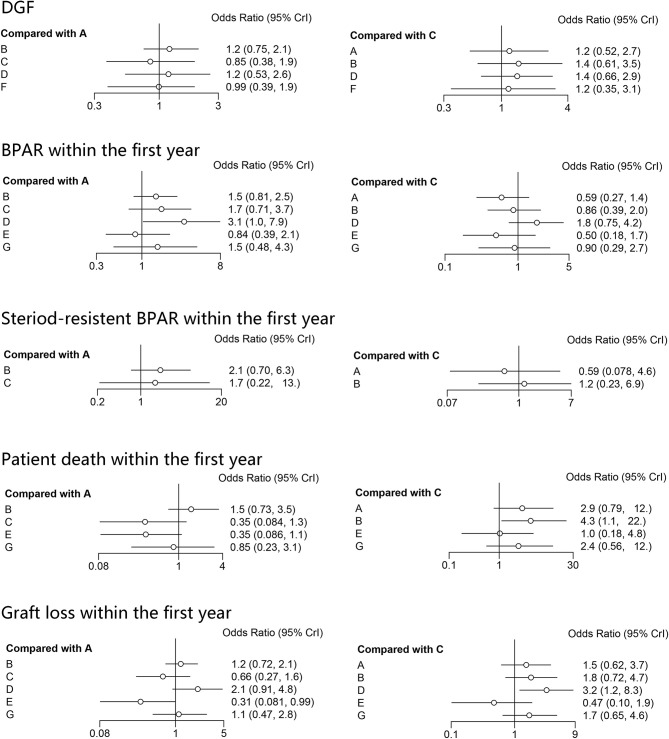
Forest plots of safety indicators. A, thymoglobulin; B, basiliximab; C, ATG-Fresenius; D, no induction therapy; E, alemtuzumab; F, daclizumab; G, OKT3.

The results of the consistency and inconsistency model results of THG vs. ATG-F in the efficacy and safety endpoints are shown in Table 3. In comparison with ATG-F, THG was not associated with a higher incidence of DGF (OR: 1.27; 95% CI: 0.53 to 2.89), patient death (OR: 2.78; 95% CI: 0.78 to 11.82), graft loss (OR: 1.40; 95% CI: 0.59 to 5.98), BPAR (OR: 0.59; 95% CI: 0.27 to 1.40), or steroid-resistant BPAR (OR: 0.61; 95% CI: 0.08 to 4.62). Similarly, no difference was found between the two in infection (OR: 1.49; 95% CI: 0.43 to 5.23), CMV infection (OR: 1.04; 95%CI: 0.22 to 4.22), *de novo* diabetes (OR: 1.10; 95% CI: 0.20 to 7.10), or malignancy (OR: 8.40; 95% CI: 0.51 to 384.61) (Figures 2, 3).

**Table 3:** Results of network meta-analyses and surface under the cumulative ranking curve (SUCRA) values.

**Outcomes**	**Study number**	**Model**	**ATG (vs. ATG-F) OR (95%CI)**	**SUCRA (THG/ATG-F)**
DGF	18	Consistency	1.27 (0.53–2.89)	0.58/0.78
		Inconsistency	1.67 (0.48–7.71)	
BPAR	22	Consistency	0.59 (0.27–1.40)	0.78/0.39
		Inconsistency	0.83 (0.22–4.85)	
Steroid-resistant BPAR	5	Consistency	0.61 (0.08–4.62)	0.76/0.49
		Inconsistency	0.54 (0.08–4.41)	
Patient survival	18	Consistency	2.78 (0.78–11.82)	0.34/0.83
		Inconsistency	2.41 (0.36–11.86)	
Graft survival	21	Consistency	1.40 (0.59–5.98)	0.59/0.83
		Inconsistency	1.12 (0.23–4.69)	
Infection	14	Consistency	1.49 (0.43–5.23)	0.54/0.79
		Inconsistency	1.32 (0.25–6.32)	
CMV infection	10	Consistency	0.96 (0.22–4.22)	0.37/0.40
		Inconsistency	1.15 (0.19–7.41)	
*De novo* diabetes	4	Consistency	2.95 (0.57–21.33)	0.30/0.90
		Inconsistency	3.12 (0.59–25.03)	
Malignancies	5	Consistency	8.33 (0.48–332.79)	0.06/0.89
		Inconsistency	7.84 (0.55–319.32)	

*ATG, anti-thymoglobulin; ATG-F, anti-thymoglobulin Fresenius; DGF, delayed graft function; BPAR, biopsy-proved acute rejection; CMV, cytomegalovirus; OR, odds ratio; 95% CI, 95% confidence intervals*.

As the SUCRA value indicated, ATG-F may be the better induction therapy than THG with regard to the prevention of DGF (78% vs. 58%, respectively), patient death (83% vs. 34%), graft loss (83% vs. 59%), infection (79% vs. 54%), CMV infection (40% vs. 37%), *de novo* diabetes (90% vs. 30%), and malignancy (89% vs. 6%). THG, however, may be better than ATG-F in preventing BPAR (78% vs. 39%) and steroid-resistant BPAR (76% vs. 49%).

Node-splitting methods were used to assess the consistency between direct and indirect results in the network meta-analysis. In general, the results regarding DGF, BPAR, patient death, graft loss, infection, and CMV infection were not significant (P > 0.05). However, significance could not be explored in steroid-resistant BPAR, *de novo* diabetes, or malignancy because no comparison was available (Table 4).

**Table 4:** Node-splitting methods to assess the corresponding degree of direct and indirect results.

**Outcome**	**Direct effect**	**Indirect effect**	**Overall**	***P*-value**
DGF	−0.70 (−2.66, 1.22)	−0.13 (−1.06, 0.87)	−0.24 (−1.06, 0.63)	0.60
BPAR	−0.61 (−3.16, 1.75)	0.67 (−0.24, 1.49)	0.52 (−0.34, 1.32)	0.32
Steroid-resistant BPAR	/	/	/	/
Patient survival	−0.63 (−3.16, 1.73)	−1.30 (−3.53, 0.43)	−1.02 (−2.47, 0.25)	0.66
Graft survival	7.96 (−1.14, 25.42)	−0.32 (−1.46, 0.51)	−0.33 (−1.09, 0.53)	0.10
Infection	0.16 (−2.38, 2.72)	−0.59 (−2.16, 0.92)	−0.40 (−1.65, 0.85)	0.57
CMV infection	−1.01 (−3.89, 1.67)	0.45 (−1.35, 2.33)	0.04 (−1.44, 1.52)	0.36
*Do novo* diabetes	/	/	/	/
Malignancies	/	/	/	/

*DGF, delayed graft function; BPAR, biopsy-proved acute rejection; CMV, cytomegalovirus*.

### Subgroup Analyses

We conducted a subgroup analysis to compare THG with ATG-F using different induction therapies—basiliximab, no induction therapy, alemtuzumab, daclizumab, and muromonab-CD3—as the intermediaries. The results are shown in Table S3. With regard to DGF, two of the three single estimates supported the overall estimates; THG seemed to be less effective in reducing the incidence of DGF when compared with ATG-F through the intermediaries of basiliximab (SUCRA values: 35% for THG, 100% for ATG-F) and no induction (SUCRA values: 65% for THG, 69% for ATG-F) but more effective through daclizumab (SUCRA values: 51% for THG, 36% for ATG-F). For graft loss, the results through the intermediaries of basiliximab (SUCRA values: 48% for THG, 81% for ATG-F) and muromonab-CD3 (SUCRA values: 43% for THG, 74% for ATG-F) supported the overall estimates. For BPAR, patient death, and infection, no difference was observed among all single and overall estimates.

We then conducted the subgroup analysis of patients at high risk for immunologic complications. We found no significant difference between THG and ATG-F with regard to the rates of DGF, patient death, graft loss, infection, CMV infection, *de novo* diabetes, malignancy, BPAR, and steroid-resistant BPAR. As the SUCRA values indicated, ATG-F may be a better choice than THG with regard to DGF (82% vs. 58%), patient death (59% vs. 35%), infection (70% vs. 23%), CMV infection (41% vs. 34%), *de novo* diabetes (90% vs. 30%), and malignancy (89% vs. 4%). THG may be a better choice than ATG-F with regard to BPAR (75% vs. 62%), steroid-resistant BPAR (85% vs. 36%), and graft loss (68% vs. 54%) (Table S4).

### Publication Bias

The network funnel plots for all nine clinical outcomes were not distinctly asymmetric. However, several trials fell outside of significance boundaries for infection, CMV infection, and BPAR analysis (Figure S2). Therefore, we conducted a subgroup analysis without those studies, which included two reports in BPAR (25, 38), one in infection (37), and three in CMV infection (17, 33, 37). Of note, THG, in comparison with ATG-F, was not associated with higher incidences of infection (OR: 1.28; 95% CI: 0.52 to 2.99), CMV infection (OR: 1.15; 95% CI: 0.34 to 4.03), or BPAR (OR: 0.54; 95% CI: 0.30 to 1.11). After we adjusted the estimates, ATG-F appeared superior to THG in preventing infection (SUCRA: 90% vs. 50%) and CMV infection (SUCRA: 43% vs. 24%), but THG seemed to be more effective than ATG-F in preventing BPAR (SUCRA: 80% vs. 32%) (Table S5).

## Discussion

To the best of our knowledge, this is the first network meta-analysis in which the efficacy and safety of THG and ATG-F were compared as induction therapies in kidney transplantation. Although we found no significant difference in safety and efficacy profiles during the first year after transplantation, ATG-F may be a better choice with regard to short-term clinical outcomes, even for patients at high risk for immunologic complications.

In comparison with basiliximab and no induction, THG and ATG-F markedly reduced the incidence of DGF. Because of the polyclonal natures of ATGs, this functional beneficial effect may not be related directly to T-cell depletion (42). Several studies have identified ATG antibodies with affinities to endovascular adhesion molecules necessary to leukocyte homing and trafficking, which indicates that ATGs may be able to reduce ischemia/reperfusion injury (43, 44). In contrast to ATG-F, THG decreased the expression of genes involved in the nuclear factor κB pathway, apoptosis, and chemoattraction even 3 months after the induction (45), which suggests that the effect of THG on inflammation may persist. However, we did not find statistical difference between THG and ATG-F in preventing DGF. The difference probably arose from the fact that ATG-F was given as a single dose, and THG was given in divided doses. Single doses may have produced a higher concentration of ATG-F, more extensively blocked adhesion between immune cells and activated endothelial cells (46), and thereby compensated for its inferiority in inflammation suppression. This hypothesis is also supported by the finding that a full induction dose of ATG-F (9 mg/kg) administrated before the reperfusion significantly decreased the occurrence of DGF (31).

THG and ATG-F have been shown to contain antibodies against several T-cell and B-cell markers, but ATG-F has a lower activity against targeted antigens and a narrower spectrum (5, 6), and THG is believed to possess more immune-modulatory and immune-inhibitory potential (45). In a retrospective study involving 342 THG-treated and 142 ATG-F-treated patients with cardiac transplants, the findings corresponded to this speculation clinically: patients who received THG had significantly fewer rejection episodes (8). A lower incidence of acute rejection after the induction treatment of THG is mainly attributed to initial lower lymphocyte counts and later recovery of lymphocyte counts in comparison with ATG-F (47, 48). By contrast, the only small-size investigation of THG and ATG-F in kidney transplantation showed similar rates of BPAR and T-cell-mediated acute rejection in the 2 year follow-up; peripheral T-cell and B-cell counts at 6 and 12 months were similar and sustained in both groups (16). Our analysis revealed comparable results between THG and ATG-F with regard to acute rejection, although SUCRA values indicated that THG might be more efficacious. T-cell recovery is dose dependent (49), but because our included studies involved various dosages for THG (1 to 8.75 mg/kg) and ATG-F (9 to 56 mg/kg), recommending comparable dosages of THG and ATG-F is difficult.

Adverse effects of immunosuppressive therapy are always of concern, especially in more intensive immunosuppressive therapies (e.g., induction treatment with ATGs). Several studies have demonstrated that viral infections, especially CMV infection, occur earlier and have a higher incidence in patients receiving an induction therapy with THG than in those receiving ATG-F (9, 50). However, we did not find any difference in CMV infection between the two groups. Viral infections are closely related to the absolute lymphocyte counts and duration of T-cell depletion after ATG induction (51, 52). Single dosing of ATG, as was performed in most of the included ATG-F studies, and immune-inhibitory potential, resulted in quicker lymphocyte, CD4, and CD8 T-cell recovery (51), which may have contributed to fewer viral infections. Of note, the patients received CMV prophylaxis with ganciclovir or valganciclovir in 73% of THG studies but in only 25% of ATG-F studies. This beneficial prophylaxis might have prevented higher rates of CMV infection after the induction treatment with THG (53).

In addition, post-transplantation malignancy occurred significantly more often and earlier in THG recipients (7, 9). CD4+ and CD8+ T cells are crucial in adaptive antiviral immunity, and antitumor effects have been mainly attributed to the activity of CD4+ T helper 1 cells, CD8+ cytotoxic T cells, and natural killer cells. Therefore, a broader spectrum of targeting cell markers expressed by various immune cells may explain the predisposition to infections and cancer complications associated with THG (54). However, we found that THG was not inferior to ATG-F with regard to the rates of malignancy. Cases of malignancy usually occurred more than 1 year after transplantation (7); thus, our study, which accounted for only the first year outcomes, may not have been sufficient to detect the difference between THG and ATG-F. In addition, although we studied other induction therapies for comparison to summarize the overall effects of ATG-F and THG, an effect of concomitant immunosuppressants may have been present.

Ourahma (55) found that the rate of graft loss was lower among the patients treated with ATG-F than among those treated with THG. Conversely, in comparison with no induction, Opelz et al. (7) reported that ATG-F was not associated with reduced renal graft loss (OR: 1.03; 95% CI: 0.9 to 1.11), whereas THG was a protective factor for graft survival (OR: 0.74; 95% CI: 0.68 to 0.81). However, because of the limited comparison and retrospective nature of these studies, these results should be interpreted with caution.

Through a network analysis of available RCTs, we found no statistical difference between THG and ATG-F, whereas ATG-F seemed to the better choice with regard to short-term patient and graft survival. The rates of acute rejection, infection, and malignancy were comparable. The long-term results might be dependent on the dosing regimen. In an observational study of 778 patients undergoing primary kidney transplantation, the intraoperative high doses of ATG-F (9 mg/kg) improved the 10 year graft survival rates than did divided-dose regimens (3 mg/kg for 7 to 8 days; 73.8% vs. 57.7%; P < 0.001) (56). Another RCT demonstrated no significant difference in patient or graft survival between a regimen of single-dose THG (6 mg/kg) and 1 of 4 alternate-day doses (1.5 mg/kg), but the single dose was associated with better renal function and patient survival (51). In our study, most of the investigators administered divided doses of THG (93.3%) or single doses or ATG-F (91.7%). Because we assessed only 1 year outcomes, whether benefits of single doses will become evident later and whether long-term outcomes will be improved are unclear.

Patients at high risk for immunologic complications experienced more rejection episodes and graft loss (57, 58). Antibody-mediated rejection accounts for a large part of acute rejection, which is usually steroid resistant (59), and it is the most common cause of allograft failure (60). THG has been shown to contain antibodies against several B-cell antigens and plasma cell-specific marker CD138; thus, THG induces B-cell apoptosis (61) and can be used to treat antibody-mediated renal allograft rejection (62). As our results indicated, THG may prevent acute rejection and steroid-resistant acute rejection more effectively and may improve graft survival in patients at high risk for complications in the first year. Because the inferiority of clinical results may not become evident until years later (63), whether the higher efficacy in acute rejection prevention will lead to more favorable long-term outcomes is uncertain.

*De novo* diabetes is largely attributed to the use of calcineurin inhibitors and steroids (64). These agents are thought to cause beta-cell depletion, and thus, minimizing the use of calcineurin inhibitors and steroids has been proposed (15, 16). However, Sánchez-Escuredo et al. (65) reported comparable 1 year outcomes of THG, ATG-F, and monoclonal antibody induction in the context of a calcineurin inhibitor-free regimen. Thomusch et al. (15) found similar rates of *de novo* diabetes in the first year among patients taking THG and basiliximab in a steroid withdrawal protocol. Although most of included studies used the common triad regimen, our pairwise and network analyses corroborated these findings as well. These limited facts indicate that THG and ATG-F might have comparable efficacy with regard to *de novo* diabetes in the short term after transplantation, but more studies with longer follow-up periods are needed to prove this conjecture.

Our study had several limitations. First, in most of the included studies, the investigators reported only short-term results; hence, we cannot compare the long-term results of ATG-F and THG. Second, the trials covered a period of 22 years, and the evolution of maintenance regimens may have introduced unmeasured differences. Finally, although we found no differences in the node-splitting analysis, the possibility of differences cannot be ignored; the comparison between THG and ATG-F is limited, and we cannot rule out the outcome assessment bias. Notably, a recent prospective, multi-center, non-interventional study (NCT03996278) has been started to compare the efficacy and safety of THG and ATG-F, which may provide more evidence for clinical practice in the future.

## Conclusions

ATG-F may be a better choice than THG in improving the short-term outcomes after kidney transplantation. Further comparison of THG and ATG-F with larger sample sizes and longer follow-up is required.

## Data Availability Statement

The datasets analyzed in this article are not publicly available due to Sichuan University data regulation policies. Requests to access the datasets should be directed to kidney5@163.com.

## Author Contributions

TS and TL: study design and paper revision. SY, XL, and YJ: data collection. TS and SY: statistical analysis and paper writing. All authors approved the submitted version of the manuscript.

### Conflict of Interest

The authors declare that the research was conducted in the absence of any commercial or financial relationships that could be construed as a potential conflict of interest.
