# Supplementary material for: Thymoglobulin vs. ATG-Fresenius as Induction Therapy in Kidney Transplantation: A Bayesian Network Meta-Analysis of Randomized Controlled Trials
Source: Front Immunol. 2020 Apr 3;11:457. doi: 10.3389/fimmu.2020.00457 (PMC7146975; doi:10.3389/fimmu.2020.00457)
Supplement: Supplementary file 1 [file Data_Sheet_1.pdf]

Supplementary Material

TABLE S1 | Immunosuppressant regimen of included studies.

Author	Arm	Immunosuppression regimen	Target level of CNI	Dose of MPA/Everolimus/AZA	Steroid	CMV prevention
Thomusch 2016[13]	Basiliximab<br>Thymoglobulin	Low-dose tacrolimus, MMF+rapid steroid withdraw	TAC: 7–12 ng/mL in first month, 6–10 ng/mL months 2-3, 3–8 ng/mL months 4–12	MMF 1 g bid on day 0 and tapering	500 mg day 0, 100 mg day 1, 75 mg day 2, 50 mg day 3, 25 mg/d days 4-7, Corticosteroids were withdrawn from day 8.	D+/R- and ATG induction patients received prophylaxis with VGC >3months
Burkhalter 2016[14]	ATG-F<br>ATG	Tacrolimus, MMF+rapid steroid withdraw	TAC:10-12 ng/ml in first month, 8-10 ng/ml months 2-3, 6-8 ng/ml months 4-6 ng/ml, 4-6 ng/ml thereafter.	MMF1g bid with the target trough level above 2 ug/ml	500 mg before IVIG, 500mg intraoperative, 500mg day 1, 250mg day 2, 0.5mg/kg/d and tapered to 0.1 mg/kg/d by month 3	D+/R-, D+/R+, D-/R+ patients received prophylaxis with VGC>3 months.
Tedesco 2015[15]	Thymoglobulin<br>Basiliximab	Tacrolimus, Everolimus, and Steriod	3-5 ng/ml<br>TAC: 3-8 ng/ml months 0-3, then reduced to 3–5 ng/ml	1.5mg bid with trough level of 4-8ng/ml	1 g intraoperative, 0.5 mg/kg/d on day 1 tapered to 5 mg/day by day 45	No prophylaxis, used preemptive
Pilch 2014[16]	Basiliximab	Tacrolimus, MMF+Steroid	TAC:6-12 ng/mL month 0-3, 5-10 ng/mL month 4-12	MMF 1 g bid on day 0 and tapering	500 mg intraoperative, 250 mg day 1, 125 mg	Prophylaxis with VGC>3 months.

	Thymoglobulin				day 2, 50 mg day 3, 20 mg days 4-30, 10 mg day 30-45, and 5 mg thereafter.	
Vanden 2013[17]	ATG-F<br>No induction	Tacrolimus, MMF+Steroid	TAC: 15-20 mg/L first 2 weeks,10-15 mg/L weeks 3-6, 5-10 mg/L thereafter	2000 mg/d week 0-2, 1500 mg/d thereafter	100 mg for the first 3 days, then tapered	D+/R-, prophylaxis with VGC
Lu 2011[18]	Alemtuzumab<br>ATG-F	Tacrolimus, MMF+Steroid	TAC: 10-13ng/ml month 0-1, 8-l0ng/ml month2-3. 6-8ng/ml month4-6, 4-6 ng/ml month 7-12	0.5g bid for <50kg, 0.75g for 50-70kg, l g for >70kg	500mg intraoperative, 8mg/kg day 0-3, then tapered	No prophylaxis
Hanaway 2011[19]	Alemtuzumab<br>Thymoglobulin	Tacrolimus, MMF+rapid steroid withdraw	TAC: 7-14 ng/ml 0-90 days, 4-12 ng/ml after 90 days	MMF 2g/d or EC-MPS 1440mg/d	Steroid withdraw by day 5	Prophylaxis with VGC
Ciancio 2010[20]	Thymoglobulin	Tacrolimus, MMF+Steroid	TAC: 6-8 ng/mL	MMF 1g bid	500 mg/d for 3 days,maintained at 0.3mg/kg at month1,then 0.15 mg/kg after 3 months	No prophylaxis
	Alemtuzumab		TAC: 4-7 ng/mL	MMF 500 mg bid	Withdraw after first week	
Noel 2009[21]	Thymoglobulin<br>Daclizumab	Tacrolimus, MMF+Steroid	TAC: 10-15 ng/ml for month 0-3, 8-12 ng/ml month 4-12	MMF 2 g/d month 1-2, 1.5g/d month 3, 1g/d thereafter	500 mg day 0, 250mg day1, 16mg/d day2-15, 12 mg/d day 16-30, 10 mg/d day 31-60, 8 mg/d day61-90, and then 0.1 mg/kg up to 1yr.	Prophylaxis with VGC 3 months.
Farney 2009[22]	Alemtuzumab<br>Thymoglobulin	CNI, MMF+Steroid	High risk: TAC 10-12 ng/mL, CsA 250-350 ng/mL in month 0-3.TAC 8-10 ng/mL, CsA 150-250 ng/mL.Low risk: TAC 8-10ng/Ml,CsA 250-325 ng/mL months0-3,Then TAC 6-8 ng/Ml, CsA 150-250 ng/mL	MMF 500mg bid for >60yr on TAC. All other MMF 1g bid,Equivalent doses of EC-MPS were used	High risk or DGF,rapid taper of steroids, achieving 5 mg/d at 2 months; All other eceived steroids for six doses only, then steroidfree	Prophylaxis with VGC>3 months.

Sheashaa 2008[23]	ATG-F<br>No induction	CNI, anti-proliferative agent+Steroid	/	/	/	/
Samsel 2008[24]	ATG-F<br>No induction	CsA+MMF(AZA)+ Steroid	CsA 8mg/kg per day in two doses	MMF 1g bid, converted to AZA 2mg/kg after 4 months	500 mg day 0, 250mg day 1-4, then 0.5 mg/kg per day	/
Kim 2008[25]	ATG-F<br>Daclizumab	CsA+MMF+ Steroid	CsA: 250–350 ng/ml months0-3, 200–250 ng/ml month4-12, and 150–200 ng/ml thereafter	MMF 1g bid with trough concentration above 2 μg/ml	0.5 mg/kg/d day 5-14,reduced by 10 mg every 2w until 30 mg/d, then by 5 mg every 2w until 15 mg/d, and thereafter by 2.5 mg until 0.1 mg/kg/d, which was kept as maintenance therapy until 6 months	/
Cantarovich 2008[26]	No induction<br>Thymoglobulin	CsA+AZA+ Steroid	CsA 150-250ng/ml	1.5mg/kg per day<br>1 mg/kg per day	2 mg/kg intraoperatively, then tapered to 5mg/day by day 90	/
Abou 2008[27]	Daclizumab<br>Thymoglobulin	CsA+MMF+ Steroid	CsA:150-250 ng/ml day7-month 2, 125-200 ng/mL months 3-6, 125-175 ng/mL months 7-12.	2 g/day	250 mg perioperatively, 1mg/kg day1-7, 0.5 mg/kg day8-14,then decreased 5mg per week until a dose of 20 mg/d,decreased by 2.5mg per week until 10 mg/d.This dose was maintained for at least 1 month and then was gradually decreased by 2.5mg per fortnight, until treatment was discontinued 5 or 6 months after transplant.	D+/R−, ganciclovir prophylaxis for 14 weeks, others receive preemptive therapy

Thomas 2007[28]	Alemtuzumab<br>Thymoglobulin	TAC+MMF+ Steroid	TAC:10ng/ml	/	250 mg intraoperative, 125 mg day1, 50 mg bid and tapered to 10 mg/d over the course of 5 days	No prophylaxis
Kyllonen 2007[29]	ATG-F<br>Basiliximab<br>No induction	CsA+AZA+ Steroid	CsA:200-300 ng/ml	2 mg/kg/d day1-2, tapering to 1 mg/kg/d on day 14	250 mg intraoperative, 40 mg/d day 1–4, tapering to 20 mg/d by day 16, and to 10–12 mg/d by 3 months	No prophylaxis
Hernandez 2007[30]	Thymoglobulin	CsA+AZA(MMF)+ Steroid	CsA: 175-300 ng/ml for first 3 months and 150-200 ng/ml thereafter	AZA 1.5 mg/kg once daily	/	ganciclovir for the first week, acyclovir for 12 weeks.
	Basiliximab		CsA: 125–175 ng/ml	MMF: 1g bid		
Brennan 2006[31]	Thymoglobulin<br>Basiliximab	TAC+MMF+ Steroid	TAC: 6-8mg/kg/d	MMF: 1g bid	7mg/kg perioperative, then tapering to 5 mg by 6 months	R+ or D+, ganciclovir prophylaxis for 3months
Ciancio 2005[32]	Thymoglobulin	TAC+MMF+ Steroid	TAC: 8-10 ng/ml	MMF: 1g bid	500mg for 3 days, tapering to 0.3 and then 0.15 mg/kg, respectively, at 1 and 3 months	Prophylaxis with VGC 3 months.
	Alemtuzumab		TAC:4 -7 ng/mL at 1 month and 4-6 ng/mL at 6 months and thereafter	MMF: 500 mg bid	Withdraw after first week	
Mourad 2005[33]	Basiliximab<br>Thymoglobulin	CsA+MMF+ Steroid	CsA: 150-200 ng/mL	MMF: 1g bid	500 mg intraoperative followed by 20 mg/d	R+ or D+, VGC prophylaxis
Tullius 2004[34]	ATG-F<br>Basiliximab	TAC+Steroid	TAC:trough level 10 ng/ml	/	500 mg perioperatively, 250 mg on day 1, tapered to 40 mg on days 2–7, then tapering	No prophylaxis
Lebranchu 2002[35]	Basiliximab	CsA+MMF+ Steroid	CsA:150±250ng/mldays0-14, 150-200ng/mL day 15-	MMF: 1g bid	250mg day 0, 1.0mg/kg days	No prophylaxis

	Thymoglobulin		week 12, 125-175ng/mL weeks 13-24		1-7, 0.5mg/kg/d days 8-14; then slowly reduced	
Yussim 2000[36]	ATG-F<br>No induction	CsA+AZA+ Steroid	/	/	/	/
Thibaudin 1999[37]	No induction<br>Thymoglobulin	CsA+AZA+ Steroid	CsA: 100-300 ng/ml	AZA 2 mg/kg/day	30 mg/day	/
Bock 1995[38]	ATG-F<br>OKT3	CsA+AZA+ Steroid	CsA:200-300ng/ml for high risk patient and 100-200ng/ml for normal patietns	AZA 2 mg/kg/day	500 mg perioperatively, 250 mg on day 1, tapered to 40 mg on days 2–7, then tapering by 5 mg every 2w until 15 mg/d, and thereafter by 2.5 mg steps thereafter	Anti-CMV globulin for prophylaxis for 18 weeks
Cole 1994[39]	Thymoglobulin<br>OKT3	CsA+AZA+ Steroid	CsA: 100-300 ng/ml	AZA 1mg/kg/day	250mg every 6h for 2days, 0.5mg/kg days3-10, 0.2mg/kg/d days11-42; then 0.15mg/kg/d	/

ATG: anti-thymoglobulin; ATG-F,anti-thymoglobulin Fresenius; CsA,cyclosporin; TAC,Tacrolimus;CMV,cytomegalovirus;MMF, mycophenolate mofetil;CNI, calcineurin inhibitor; AZA, acetazolamide;VGC, valganciclovir

TABLE S2 | Quality evidence assessment of 27 included studies. “1” = low risk; “/” = unknown; “0” = high risk.

Terms	Thomusch 2016	Burkhalter 2016	Tedesco-Silva 2015	Pilch 2014	van den Hoogen 2013	Lu 2011	Hanaway 2011	Ciancio 2010	Noel 2009	Farney 2009	Sheashaa 2008	Samsel 2008	Kim 2008	Cantarovich 2008	Abou-Ayache 2008	Thomas 2007	Kyllonen 2007	Hernandez 2007	Brennan 2006	Ciancio 2005	Mourad 2004	Tullius 2003	Lebranchu 2002	Mourad 2001	Yussim 2000	Thibaudin 1998	Bock 1995	Cole 1994
Random sequence generation (selection bias)	1	1	1	1	1	1	1	1	1	1	1	1	1	/	1	1	1	1	1	1	1	/	1	1	/	1	/	1
Allocation concealment (selection bias)	1	1	1	/	1	/	1	/	1	1	1	/	/	/	1	1	1	1	1	1	/	/	1	1	/	/	/	1
Masking of participants and personnel (performance bias)	1	1	1	/	1	/	1	/	/	1	1	/	/	/	1	/	1	0	1	/	/	/	/	1	/	/	/	0
Masking during outcome assessment (detection bias)	1	0	/	/	/	/	1	/	/	/	0	/	/	/	/	/	/	/	1	/	/	/	/	0	/	/	/	/
Incomplete outcome data (attrition bias)	1	1	1	1	1	/	1	/	1	1	0	1	1	1	1	0	1	1	1	1	1	0	1	1	0	1	1	1
Selective reporting (reporting bias)	1	1	1	1	1	1	1	/	1	1	/	/	1	1	1	1	1	1	1	1	1	/	1	1	1	1	1	1

TABLE S3 | Subgroup analyses using different induction therapies as the intermediary.

Outcomes	Study (N)	Model	Via Basiliximab OR (95%CI)	SUCRA (THG/ATG-F)	Study (N)	Via no induction OR (95%CI)	SUCRA (THG/ATG-F)	Study (N)	Alemtuzumab OR (95%CI)	SUCRA (THG/ATG-F)	Study (N)	Daclizumab OR (95%CI)	SUCRA (THG/ATG-F)	Study (N)	OKT3 OR (95%CI)	SUCRA (THG/ATG-F)
DGF	8	Consistency	3.45 (1.20-10.31)	0.35 / 1.00	6	1.04 (0.31-3.55)	0.65 / 0.69	/	/	/	6	0.76 (0.09-5.56)	0.51 / 0.36	/	/	/
		Inconsistency	2.79 (0.73-10.67)			1.14 (0.30-4.68)			/			0.93 (0.11-7.55)			/	
BPAR	10	Consistency	0.64 (0.32-1.38)	0.92 / 0.25	5	0.61 (0.07-5.05)	0.78 / 0.55	7	0.80 (0.19-3.59)	0.43 / 0.33	/	/	/	3	0.30 (0.08-1.37)	0.91 / 0.13
		Inconsistency	0.65 (0.26-1.66)			0.63 (0.07-5.60)			0.80 (0.16-3.64)			/			0.35 (0.08-1.69)	
Steriod-resistent BPAR	5	Consistency	0.60 (0.08-4.55)	/	/	/	/	/	/	/	/	/	/	/	/	/
		Inconsistency	0.60 (0.09-4.68)			/			/			/			/	
Patient death	10	Consistency	4.45 (0.65-50.23)	0.45 / 0.96	/	/	/	7	1.56 (0.30-9.13)	0.25 / 0.64	/	/	/	3	2.13 (0.45-11.29)	0.30 / 0.82
		Inconsistency	3.82 (0.48-54.38)			/			1.74 (0.27-10.94)			/			2.21 (0.45-11.40)	
Graft loss	9	Consistency	1.69 (0.40-7.83)	0.48 / 0.81	7	0.38 (0.06-1.89)	0.94 / 0.39	5	0.21 (0.01-2.93)	0.71 / 0.13	/	/	/	3	1.68 (0.17-11.20)	0.43 / 0.74
		Inconsistency	1.67 (0.23-8.77)			0.37 (0.07-2.27)			0.18 (0.00-3.42)			/			1.46 (0.11-13.21)	
Infection	9	Consistency	1.76 (0.44-6.68)	0.19 / 0.77	/	/	/	5	1.45 (0.09-21.91)	0.51 / 0.66	/	/	/	3	1.04 (0.22-5.24)	0.64 / 0.67

		Inconsistency	1.47 (0.24-7.36)			/			1.37 (0.09-19.53)			/			1.02 (0.21-5.29)	
CMV infection	10	Consistency	0.96 (0.22-4.22)	/	/	/	/	/	/	/	/	/	/	/	/	/
		Inconsistency	1.15 (0.19-7.41)			/			/			/			/	
De novo diabetes	4	Consistency	2.95 (0.57-21.33)	/	/	/	/	/	/	/	/	/	/	/	/	/
		Inconsistency	3.12 (0.59-25.03)			/			/			/			/	
Malignancies	5	Consistency	8.33 (0.48-332.79)	/	/	/	/	/	/	/	/	/	/	/	/	/
		Inconsistency	7.84 (0.55-319.32)			/			/			/			/	

TABLE S4 | Subgroup analyses of studies enrolling patients with immunologically high risk.

Outcomes	Study number	Model	THG (vs ATGF) OR (95%CI)	SUCRA (THG/ATG-F)
DGF	8	Consistency	1.66 (0.40-5.94)	0.58 / 0.82
		Inconsistency	1.81 (0.37-8.47)	
BPAR	9	Consistency	0.86 (0.33-2.40)	0.75 / 0.62
		Inconsistency	0.92 (0.27-3.47)	
Steriod-resistent BPAR	2	Consistency	0.22 (0.00-16.66)	0.85 / 0.36
		Inconsistency	0.21 (0.00-18.12)	
Patient death	7	Consistency	1.93 (0.23-17.18)	0.35 / 0.59
		Inconsistency	1.95 (0.21-18.26)	
Graft loss	9	Consistency	0.82 (0.16-4.57)	0.68 / 0.54
		Inconsistency	0.78 (0.10-4.62)	
Infection	4	Consistency	1.52 (0.46-4.22)	0.23 / 0.70
		Inconsistency	1.42 (0.40-4.79)	
CMV infection	4	Consistency	1.01 (0.22-4.86)	0.34 / 0.41
		Inconsistency	1.23 (0.20-4.95)	
De novo diabetes	0	Consistency	2.95 (0.57-21.33)	0.30 / 0.90
		Inconsistency	3.12 (0.59-25.03)	
Malignancies	3	Consistency	21.31 (0.77-1421.34)	0.04 / 0.89
		Inconsistency	22.73 (0.74-1413.75)	

TABLE S5 | Subgroup analyses excluding sensitive studies observed in the funnel plots.

Outcomes	Study number	Model	ATG (vs ATG-F) OR (95%CI)	SUCRA (THG/ATG-F)
BPAR	9	Consistency	0.54 (0.30-1.11)	0.80 / 0.32
		Inconsistency	0.79 (0.24-3.51)	
Infection	4	Consistency	1.60 (0.73-3.16)	0.50 / 0.90
		Inconsistency	1.33 (0.40-3.54)	
CMV infection	7	Consistency	1.15 (0.34-4.03)	0.24 / 0.43
		Inconsistency	1.35 (0.31-4.51)	

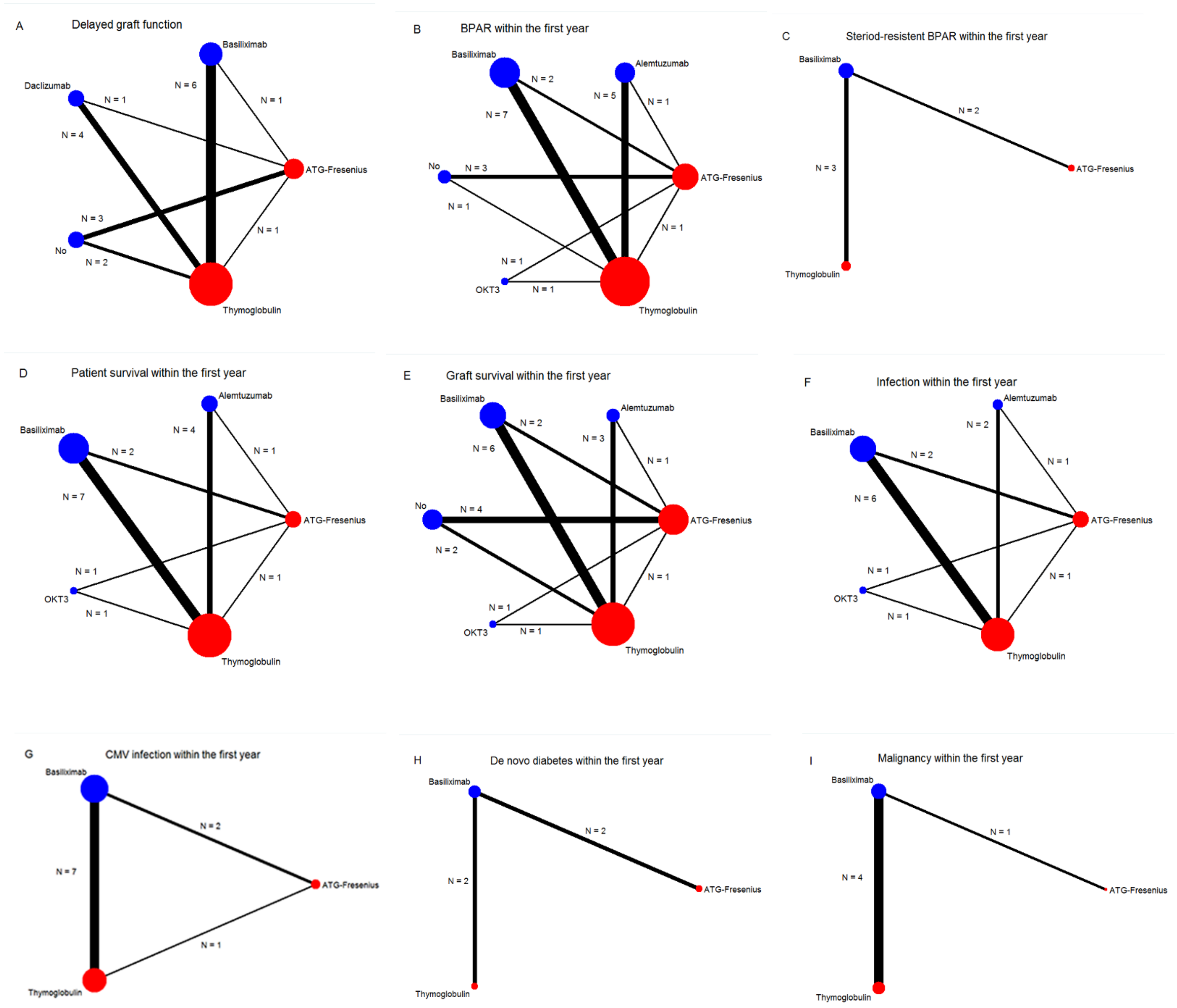

FIGURE S1 | Network of eligible comparisons for efficacy and safety of induction therapies. A, thymoglobulin; B, basiliximab; C, ATG-Fresenius; D, no induction therapy; E, alemtuzumab; F, daclizumab; G, OKT3.

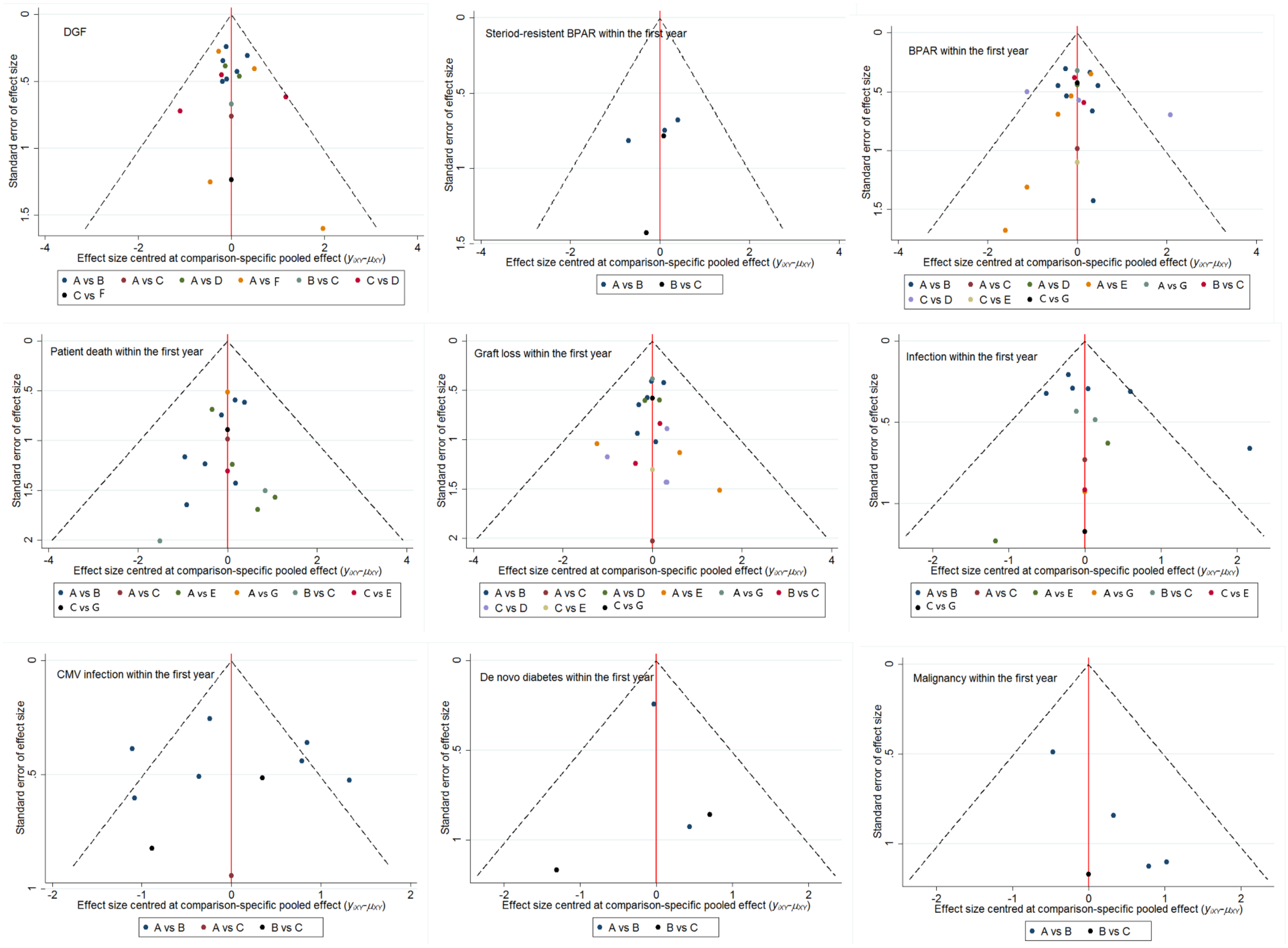

FIGURE S2 | Funnel plots for efficacy and safety of induction therapies. A, thymoglobulin; B, basiliximab; C, ATG-Fresenius; D, no induction therapy; E, alemtuzumab; F, daclizumab; G, OKT3.
